# Clinical Reality and Outcomes of Home Semi-Solid Enteral Nutrition Guidance and Management Using a Medical Claims Database in Japan: A Retrospective Cohort Study

**DOI:** 10.3390/nu18152442

**Published:** 2026-07-27

**Authors:** Mio Matsuoka, Hiroko Shimaya, Yoshihito Iwanami, Shohei Iijima

**Affiliations:** 1 Department of Nutrition, Osaka International Cancer Institute, Osaka Prefectural Hospital Organization, Osaka 541-8567, Japan; mio.matsuoka@oici.jp; 2 Department of Nutrition Oncology, Osaka International Cancer Institute, Osaka Prefectural Hospital Organization, Osaka 541-8567, Japan; 3 R&D Application Office, Research and Development Division, EN Otsuka Pharmaceutical Co., Ltd., Tokyo 101-0052, Japan; shimaya.hiroko@otsuka.jp (H.S.); iwanami.yoshihito@otsuka.jp (Y.I.)

**Keywords:** semi-solid enteral nutrition, gastrostomy, hospital stay, pneumonia, medical claims database, real-world data

## Abstract

Background/Objectives: The semi-solid enteral nutrition method, in which semi-solid nutrients are administered through a gastrostoma rather than liquid nutrients, is a medical technology for nutritional management developed in Japan. This nutritional method has spread rapidly, and in 2018, the Home Semi-solid Enteral Nutrition Guidance and Management fee was newly established in the payment system for medical services. However, there are few studies evaluating this nutrition method in actual clinical practice. Methods: Using the DeSC medical claims database, we investigated patient characteristics, the number of fee calculations, and the length of hospital stay for all hospitalizations and for pneumonia during and after fee calculation. Results: Home semi-solid enteral nutrition guidance and management was received at least once by 3105 patients (mean age: 79.4 years). Cerebrovascular disease, nervous system disease, dementia, and pneumonia were the most common diseases at the start of guidance and management, and the mean number of sessions received was 7.7. The mean length of stay for any hospitalization was significantly shorter during the guidance and management period than after the last guidance and management session (8.2 vs. 29.1 days). Notably, the mean length of hospital stay for pneumonia was also significantly shorter during the guidance and management period than after the last guidance and management session (3.1 vs. 10.2 days). Conclusions: This study clarified the calculation status of the Home Semi-solid Enteral Nutrition Guidance and Management fee and outcomes of patients who received this guidance and management. These findings suggest that the provision of this guidance and management may be associated with the shorter length of hospital stay. However, because residual confounding cannot be ruled out, a causal relationship cannot be established. Overall, the provision of this guidance and management may be associated with maintaining stable home care.

## 1. Introduction

Enteral nutrition is a method of providing nutritional support via the gastrointestinal tract for patients with impaired oral intake. Gastrostomy may be performed if enteral feeding is needed. However, liquid enteral feeding through the gastrostoma may cause several complications, such as gastroesophageal reflux, reverse leakage of nutrients from the gastrostoma, and diarrhea [1,2]. To address these problems, the semi-solid enteral nutrition (SEN) method, which uses semi-solidified nutrients instead of liquid nutrients, has been developed in Japan [3] for nutritional management. A meta-analysis demonstrated that compared with conventional liquid nutrients, SEN significantly reduces the risk of gastroesophageal reflux and dwell time in the stomach and also shortens care time, such as the time needed to prepare and administer nutritional treatments [4]. Additionally, the benefits of using SEN have been reported, such as a reduced incidence of pneumonia [5] and diarrhea [6]. However, in some reported cases, SEN was not appropriately continued after the transition to home care [7]. Therefore, appropriate guidance and management are considered essential to using SEN safely and continuously at home. Against this background, the Home Semi-solid Enteral Nutrition Guidance and Management (HSEN) fee was newly established in the fiscal year (FY) 2018 revision of Japan’s payment system for medical services.

The HSEN fee is established under the uniform national medical fee schedule. Regulations for the HSEN fee require that guidance and management be provided for non-hospitalized patients and their families, addressing not only the SEN method itself but also the restoration of oral intake. In other words, the HSEN fee does not only refer to the use of semi-solid nutrients. The HSEN method aims to help patients achieve good oral intake level through safe and effective enteral nutrition. Multifaceted care is required, depending on patients’ swallowing function and living environment, as well as semi-solid nutritional management. HSEN is designed to contribute to improvement in the quality of life of at-home patients with a gastrostoma. Regarding guidance and management provided during hospitalization for transitioning to home care, initial calculation of the HSEN fee is performed on the discharge date. There is a time limit for the calculation of the HSEN fee, namely, 12 months from the month of the first calculation. This is because the HSEN fee places importance on supporting appropriate nutritional management and the transition to oral intake at an early stage after gastrostomy. According to NDB (National Database of Health Insurance Claims and Specific Health Checkups of Japan) open data released by the Japanese Ministry of Health, Labour and Welfare, the number of HSEN fee calculations has increased each year, rising from 26,369 in 2018 [8] to 52,622 in 2023 [9]. However, even 7 years after the establishment of this system, there are few reports from large-scale, multicenter studies on the clinical reality regarding this method and fee calculation, such as the background of patients for whom HSEN fees were calculated, guidance that was actually provided, and patient outcomes. Considering these circumstances, we aimed to descriptively clarify the clinical reality regarding the HSEN method and the outcomes of patients who received HSEN in the context of home nutritional management in Japan. Our findings will contribute to discussions regarding international development of the SEN method.

Database research can be performed with minimal cost and time [10]. Therefore, in recent years, many studies using real-world data have been performed in Japan [11]. Medical insurance in Japan involves a universal health insurance system, and all citizens are enrolled in this system, in principle. The DeSC database is a commercial health care database. As of March 2023, the DeSC included registered data for approximately 12 million people covering all age groups, including information on medical claims and specific health checkups related to the universal health insurance system [12]. The sex and age distributions of the population included in this database are similar to estimates based on the national population census [13]. According to open data of the 10th NDB [9], approximately 86% of HSEN fees are calculated for patients aged 65 years or older. Using the DeSC database, which contains data comparable to population estimates and includes a large amount of information on older patients, we aimed to describe the clinical characteristics and outcomes of patients who received HSEN during and after the period of guidance and management.

## 2. Materials and Methods

### 2.1. Databases

This study was performed using the DeSC database, provided by DeSC Healthcare, Inc. (Tokyo, Japan). The HSEN fee is a nationally standardized reimbursement item under Japan’s universal health insurance fee schedule. However, because this study used claims data, detailed information on the specific content of guidance and management, the healthcare professionals involved, and whether the service was provided at the request of the patient/family, in urgent situations, or at predetermined intervals was not available in the database. In routine clinical practice, these aspects are determined by the attending physician according to each patient’s clinical condition and home-care environment. Because regulations for the HSEN fee stipulate that SEN at home should be initiated within 1 year after gastrostomy, we used the claims data of patients who had undergone gastrostomy from April 2017 (1 year before establishment of the HSEN fee) to August 2022.

### 2.2. Study Populations

In the present study, patients for whom HSEN fees were calculated were considered patients who had received HSEN.

To clarify the observation period for each patient, we defined the first and last HSEN dates as follows: the HSEN start date was the day on which a HSEN session was first provided, and the final HSEN date was the last day on which HSEN was provided within +365 days from the start date.

**Figure 1:**
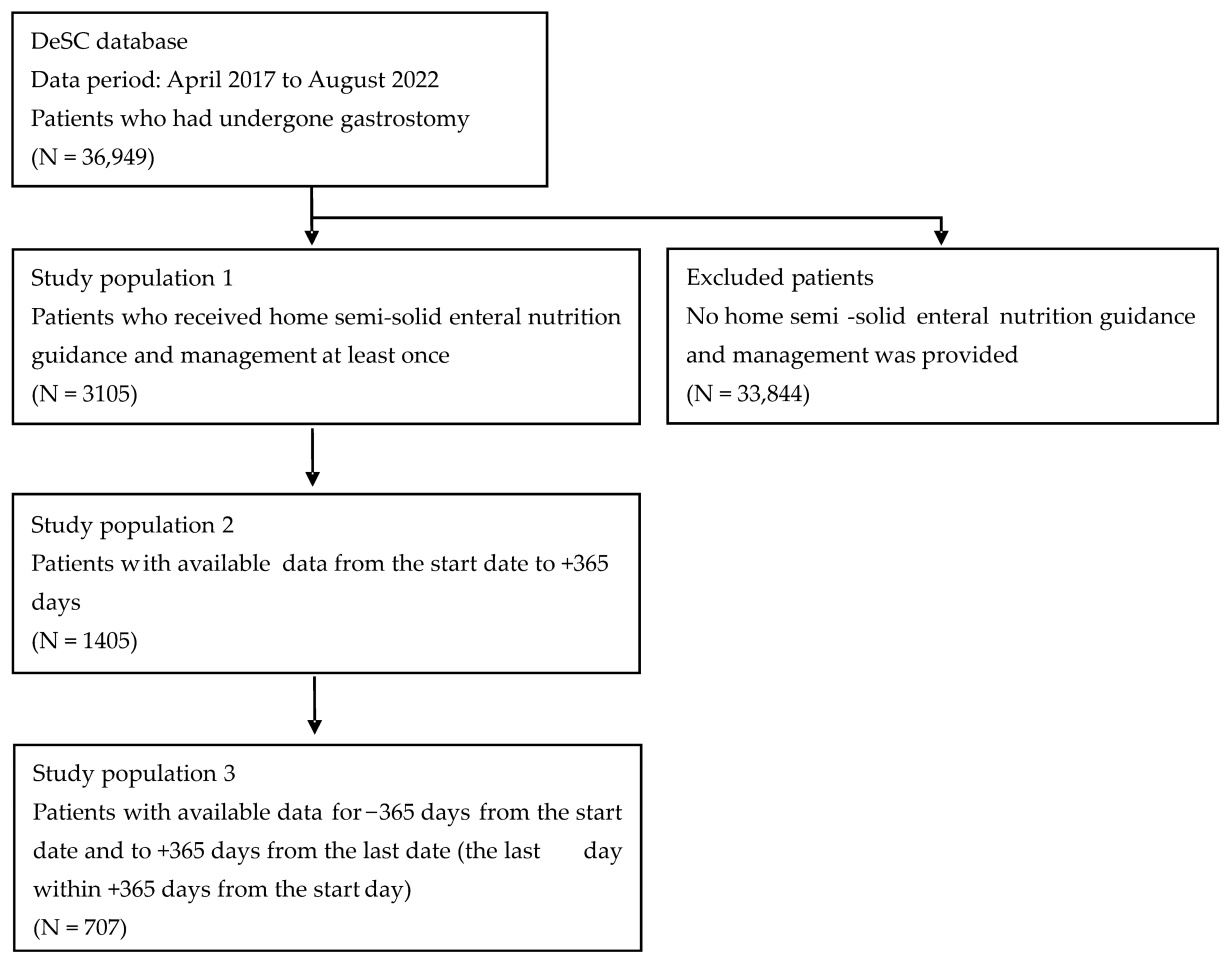
Flowchart of the three study populations. Among patients who had previously undergone gastrostomy, we excluded patients who did not receive home semi-solid enteral nutrition guidance and management.

We established three study populations to characterize patients who received HSEN. Study population 1 was defined as patients who received HSEN at least once. Study population 2 was defined as patients in Study population 1 who had data +365 days from the HSEN start date, to clearly establish the end date of the HSEN provision period. Study population 3 was defined as patients in Study population 2 who had data −365 days from the HSEN start date and +365 days from the last HSEN date, to investigate patient characteristics before the first HSEN session and after the final HSEN session (Figure 1).

### 2.3. Outcomes

In this study, diseases were defined using International Statistical Classification of Diseases and Related Health Problems 10th Revision (ICD-10) codes, as follows: malignant neoplasms of lip, oral cavity, and pharynx (C00–C14); malignant neoplasms of digestive organs (C15–C26); other malignant neoplasms (C30–C41, C43–C58, and C60–C97); amyotrophic lateral sclerosis (G122); Parkinson’s disease (G20); epilepsy (G40–G41); multiple system atrophy (G903); other diseases of the nervous system (G11, G13–G14, G21–G22, G24–G26, G32, G35–G37, and G70–G73); bacterial pneumonia (J15); pneumonia, organism unspecified (J18); pneumonitis due to solids and liquids (J69); other pneumonia (J12–J14 and J16–J17); dementia (F00–F03); cerebrovascular disease (I60–I69); respiratory failure (J96); severe motor and intellectual disabilities (F791); and cerebral palsy (G80). These definitions were based on classifications and codes developed through discussions among multidisciplinary experts during our previous small-scale registry study involving a similar population. Pneumonia was defined using ICD-10 codes J12–J18 and J69. However, due to the nature of this database, it was not possible to differentiate between acute concomitant pneumonia, recurrent aspiration pneumonia, and aspiration-associated pneumonia. Drug data were extracted using computerized claims processing system drug codes. Data for medical procedures were extracted using computerized claims processing system medical care activity codes for medical care activities.

### 2.4. Statistical Analysis

This study was designed as a descriptive analysis of claims data and was not intended to evaluate the causal effect of HSEN or to compare incidence between exposed and unexposed groups. Statistical analysis services for this study were contracted to A2 Healthcare Corporation (Tokyo, Japan). Summary statistics were calculated for continuous data on patient characteristics, and frequency counts (number of patients and percentage in each study population) were calculated for categorical data.

Supplementary Tables S1 and S2 were intended as descriptive summaries of patient characteristics and reasons for hospitalization, rather than for formal hypothesis-driven comparisons between mutually exclusive groups. Therefore, inferential statistical testing was not performed for these tables, and post-hoc multiplicity correction was not applied.

Regarding the length of hospital stay, we calculated summary statistics for the length of stay and for changes in the length of hospital stay.

Normality of the continuous variables was assessed using the Shapiro–Wilk test. As all variables significantly deviated from normality, the Wilcoxon signed-rank test was used for paired non-parametric comparisons within the same patients.

Taking into account variations in the length of observation periods, a similar analysis was performed with values calculated per month of observation period for hospital stays. A two-sided *p* value < 0.05 was considered statistically significant.

All statistical analyses were performed using SAS version 9.4 (SAS Institute Inc., Cary, NC, USA). Because this was a retrospective database study including all eligible patients during the predefined study period, no a priori sample size calculation or post hoc power analysis was performed.

## 3. Results

We investigated the characteristics of patients who had received HSEN at least once (Study population 1). This study population comprised 3105 patients: 1576 men (50.8%) and 1529 women (49.2%), with a mean (±standard deviation) age of 79.4 ± 11.5 years. Diseases at the first HSEN included cerebrovascular diseases in 1249 patients (40.2%), nervous system diseases in 1162 patients (37.4%), dementia in 1149 patients (37.0%), and pneumonia in 878 patients (28.3%). The facilities in which gastrostomy was performed were a hospital in 3078 patients (99.1%) and a clinic in 18 patients (0.6%). In Japan, the Medical Care Act defines hospitals as medical institutions with 20 or more beds and clinics as those with fewer than 20 beds or no inpatient beds. For 704 (22.7%) patients, another evaluation related to swallowing function at the time of gastrostomy was also performed (Table 1).

**Figure 2:**
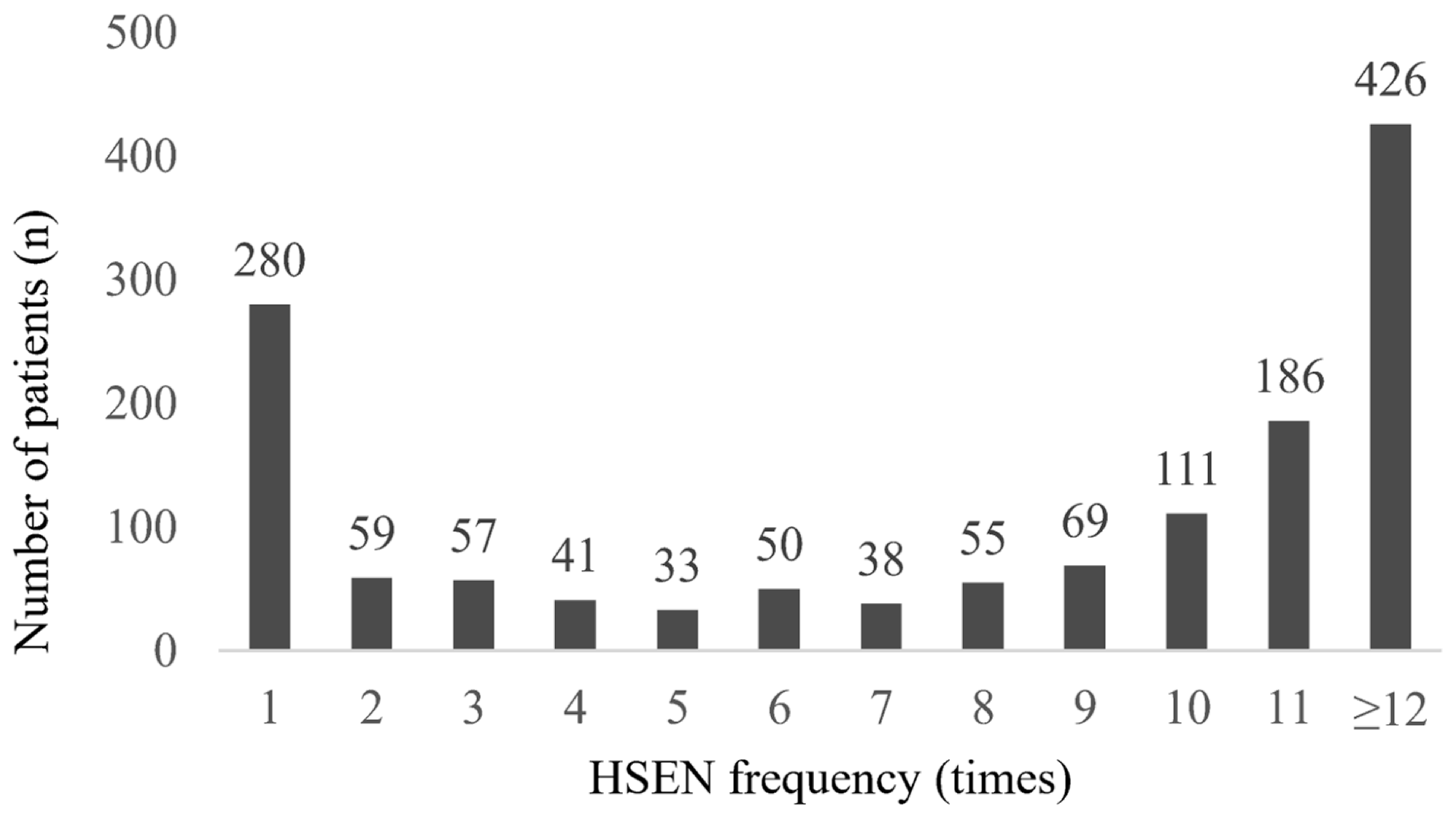
Number of home semi-solid enteral nutrition guidance and management sessions. The graph shows the number of patients according to the number of times that home semi-solid enteral nutrition guidance and management was provided. HSEN, Home Semi-solid Enteral Nutrition Guidance and Management.

Patient characteristics during the period in which they received HSEN were investigated in Study population 2, which included 1405 patients. A total of 426 patients (30.3%) had 12 or more treatments with the HSEN method, 280 patients (19.9%) had one treatment, 33 patients (2.3%) had five, and 38 patients (2.7%) had seven treatments; the distribution of the data was U-shaped (Figure 2). The mean number of sessions received was 7.7 ± 4.5. The medical institutions that most often provided HSEN were clinic–clinic (first–second calculation) in 719 patients (51.2%), followed by hospital–hospital in 257 patients (18.3%). The medical institutions associated with patients who received HSEN only once were hospitals for 237 patients (16.9%) and clinics for 45 patients (3.2%) (Table 2). Regarding medical care received during the period in which patients were receiving HSEN, semi-solid nutritional products approved for pharmaceutical use were prescribed for 1202 patients (85.6%), three patients (0.2%) underwent measurement of serum selenium levels, three patients (0.2%) received home-visit rehabilitation guidance and management for home-care patients, 520 patients (37.0%) received home-visit dental care, 24 patients (1.7%) received home-visit dental hygiene guidance, and 15 patients (1.1%) received home-visit oral rehabilitation guidance and management for home-care patients. During the period in which HSEN was being provided, five patients (0.4%) died (Table 3).

We investigated patient characteristics after the last HSEN session in Study population 3, which included 707 patients. Regarding medical care after the last HSEN session, semi-solid nutritional products approved for pharmaceutical use were prescribed for 581 patients (82.2%), seven patients (1.0%) had measurement of serum selenium levels, one patient (0.1%) received home-visit rehabilitation guidance and management for home-care patients, 323 patients (45.7%) received home-visit dental care, 40 patients (5.7%) received home-visit dental hygiene guidance, and 15 patients (2.1%) received home-visit oral rehabilitation guidance and management for home-care patients. After the last HSEN session, 12 patients (1.7%) died (Table 4).

Because HSEN was provided to patients with various diseases, we investigated the characteristics of each disease. However, there was no notable difference among diseases (Supplementary Table S1 and Figure S1).

To identify patient outcomes after the last HSEN session, we investigated the reasons for patient hospitalization and total length of hospital stay, as well as the length of hospital stay for pneumonia, during the period in which HSEN was provided and after the last HSEN session. A total of 224 patients were hospitalized during the HSEN provision period. The reasons for hospitalization were pneumonia in 96 patients (42.9%), nervous system diseases in 91 patients (40.6%), and cerebrovascular diseases in 82 patients (36.6%). A total of 340 patients were hospitalized after the last HSEN session. The reasons for hospitalization were pneumonia in 194 patients (57.1%), nervous system diseases in 143 patients (42.1%), and cerebrovascular diseases in 141 patients (41.5%) (Supplementary Table S2). The total length of hospital stay was significantly shorter during the HSEN provision period (8.2 ± 21.3 days) than after the last HSEN session (29.1 ± 64.0 days). Considering variations in the length of the observation period, the total length of hospital stay per month of observation period was calculated; this was significantly shorter during the HSEN provision period (1.74 ± 5.25 days) than after the last HSEN session (2.39 ± 5.26 days).

Examination of hospitalizations for pneumonia showed that the length of hospital stay was significantly shorter during the period in which HSEN was provided (3.1 ± 10.8 days) than after the last HSEN session (10.2 ± 25.4 days). The length of hospital stay for pneumonia per month of observation was significantly shorter during the HSEN provision period (0.58 ± 2.74 days) than after the last HSEN session (0.84 ± 2.09 days) (Table 5).

## 4. Discussion

The present study was the first to investigate the association between patient outcomes and the HSEN method using real-world data from a Japanese medical claims database. According to a report by Okuyama et al. [14] and a special investigation verifying results of the FY 2014 medical fee revision by the Japanese Ministry of Health, Labour and Welfare (a survey of the implementation of gastrostomy and other procedures) [15], most patients were older at the time of gastrostomy, and the reasons for gastrostomy were mainly cerebrovascular disease, aspiration pneumonia, and dementia. These findings are similar to those among participants in the present study. There was also no notable difference in characteristics according to disease, suggesting that the current requirements for the HSEN are not limited to certain diseases or age groups in patients with gastrostoma and that the HSEN is applicable to a broad range of patients.

We determined the number of HSEN sessions and outcomes after the last HSEN session. Previous studies on the HSEN fee examined in-hospital systems meeting the calculation requirements [16] and the number of patients for whom the fee was actually calculated [17]. These reports had limited information on the distribution of the number of HSEN sessions per case or on patient outcomes after the last HSEN session. The results of our study showed that the distribution of the number of HSEN sessions per case was U-shaped, and the highest proportion of patients had 12 or more sessions; the next highest proportion of patients had one or two sessions, indicating polarized data. This suggests that maintaining long-term guidance can be difficult in home-care settings, potentially due to challenges in care continuity, limited hospital–clinic coordination, and the burden on patients and caregivers. Discontinuation of HSEN billing does not necessarily indicate the end of nutritional support, but may reflect the end of formal guidance within the reimbursement period. Patients in the ≥12 sessions group may reflect extended support due to clinical complexity or difficulties in transitioning to stable home care, although directly comparable evidence is limited due to the novel nature of this research area. To address these bottlenecks, stronger discharge planning, improved inter-facility collaboration, and more flexible, patient-tailored follow-up should be considered.

We investigated changes in the length of hospital stay during the period in which HSEN was provided and after the last HSEN session and found that the total length of hospital stay was shorter during the HSEN provision period than that after the last session. Moreover, when limited to hospital stays for pneumonia, this duration was also shorter during the HSEN provision period than the duration after the last HSEN session.

Regulations for the HSEN fee necessitate that oral care, eating and swallowing training, and other activities be carried out to restore oral intake. It has been reported that oral care management leads to a reduced risk of pneumonia [18,19] and that eating and swallowing training reduces the incidence of pneumonia [20,21]. Previous studies targeting older patients receiving home care have suggested that multidisciplinary home care may be associated with reduced hospital utilization [22,23]. Based on these findings, the provision of this multidisciplinary guidance and management in the present study may have influenced patterns of healthcare utilization. Although oral intake status cannot be evaluated in detail using analysis of claims data because of the nature of such data, results regarding the shorter length of hospital stay for pneumonia suggest that nutritional management using the SEN method and multifaceted multidisciplinary care, including oral care management together with eating and swallowing training—which are required to calculate the HSEN fee—may be associated with maintaining stable home care. However, further studies are needed to examine these associations, including their causal relationships.

We found that selenium levels in the blood were rarely measured during HSEN provision and after the last HSEN session. Selenium is an essential trace element, and a normal diet does not lead to selenium deficiency. However, it has been reported that prolonged enteral nutrition using supplements containing little selenium may cause selenium deficiency [24]. Deficiency in this trace element causes cardiomyopathy, arrhythmia, susceptibility to infection, anemia, muscle weakness, and even death in serious cases. However, selenium levels were rarely measured in the present study, and semi-solid nutrients prescribed during the study period contained little selenium. Therefore, attention should be paid to regular monitoring of serum selenium levels during long-term enteral nutrition. After the end of this study, a novel semi-solid nutrient preparation containing the recommended amounts of selenium became available for clinical use. In the future, the use of such products should be considered.

This study revealed the clinical reality that in Japan, the HSEN is mainly applied for patients with primary diseases such as cerebrovascular disease, nervous system disease, and dementia. However, according to guidelines of the European Society for Clinical Nutrition and Metabolism, gastrostomy is indicated for long-term nutritional management in patients with head and neck cancer, oral cancer, dysphagia, and for older people [25]. Newly performing gastrostomy is not recommended for patients with severe dementia [26]. Regarding diarrhea, which is a common problem with the administration of liquid nutrients, nutrient compositions containing dietary fiber have been proposed, but the formulation of nutrients such as semi-solid ones is not mentioned [25]. On this basis, it is surmised that liquid nutrients are mainly used in other countries, as reports of semi-solid nutrients are extremely limited. In addition, the HSEN examined in this study represent a comprehensive care approach that includes not only the effects of the SEN method itself but also those of the multiple intervention components described above. Therefore, the findings of this study are more appropriately interpreted as reflecting the potential effects of a multifaceted intervention rather than those of the SEN method alone. However, the findings of this study, which were based on real-world data from Japan, may not be directly or readily generalizable to clinical practice in other countries. This is because healthcare systems, clinical settings, and resources related to oral care vary across countries, and the quality and extent of such multifaceted interventions may also vary and should be carefully considered.

This study has several limitations. First, this was a descriptive retrospective claims database study and was not designed to establish causality or evaluate the treatment effect of HSEN. In addition, the DeSC database lacks sufficiently detailed clinical information, making construction of an appropriate external control group difficult. Second, because this study compared outcomes during the HSEN period with those after the final HSEN calculation, patients without an evaluable post-HSEN observation period could not be included in analyses requiring such follow-up. Therefore, these findings should be interpreted as descriptive observations of the clinical reality of HSEN in Japan rather than as evidence of causal clinical benefit. Furthermore, although the DeSC database was useful for identifying the implementation of HSEN and events such as hospitalization, it did not allow for a detailed evaluation of the indications for HSEN initiation or the underlying clinical characteristics of patients. In particular, clinically important factors, including aspiration risk, nutritional status, the severity of sarcopenia and frailty, activities of daily living, and the caregiving environment, were not available. It was also not possible to determine the specific content of the guidance and management provided to individual patients, the healthcare professionals involved, or whether the timing of implementation was planned or unplanned. Therefore, the possibility that these unmeasured confounding factors influenced the results of this study cannot be excluded, and the findings should be interpreted with caution. It should also be noted that the data alone were insufficient to accurately ascertain the clinical reality of home care and outcomes for patients receiving HSEN. We investigated the implementation of guidance and management to restore oral intake during and after the HSEN provision period; however, home rehabilitation guidance and management as well as home-visit dental hygiene guidance were rarely calculated. This is because many participants in this study were aged 65 years or older and may have been certified as requiring long-term care; thus, services received by these patients that carried these guidance and management fees may have been covered by long-term care insurance rather than medical insurance. Therefore, to investigate home care status in detail, the calculation status of corresponding long-term care insurance fees should also be determined. However, the DeSC database used in this study does not include data on long-term care insurance; therefore, we could not determine the use of that insurance among our study populations. This may have resulted in only a partial understanding of the actual care provided in the home setting and represents an important limitation in interpreting the findings of this study.

We defined participants during the HSEN provision period as patients who had data +365 days from the start date of HSEN, and we defined participants after the last HSEN session as patients who had data +365 days from the date of the last session. Therefore, patients who died during the HSEN provision period or within +365 days from the last HSEN session were excluded from the study. Thus, the actual number of patients who received HSEN at least once and who died may be higher than the number obtained in this study.

Given these considerations, further studies, particularly prospective registry and cohort studies, are needed to clearly evaluate the effectiveness of HSEN while appropriately accounting for these factors.

## 5. Conclusions

This study was conducted using the DeSC database to clarify the actual status of HSEN and patient outcomes during the period in which HSEN was provided and after the last HSEN session. The results showed that most patients who received HSEN were aged 65 years or older. Cerebrovascular disease, nervous system disease, and dementia were the most common diseases at the initiation of HSEN. Moreover, the total length of hospital stay and length of hospital stay for pneumonia were significantly shorter during the HSEN provision period than after the last HSEN session. These findings suggest that HSEN may be associated with maintaining stable home care. However, the possibility of residual confounding and confounding by indication cannot be completely ruled out.

## Figures and Tables

**Table 1:** Patient background before the start of home semi-solid enteral nutrition guidance and management (N = 3105).

			**n (%)**
Sex	Male		1576 (50.8)
	Female		1529 (49.2)
Age (years)	Mean [standard deviation]		79.4 [11.5]
Disease	Malignant neoplasm		593 (19.1)
		Malignant neoplasms of lip, oral cavity and pharynx	124 (4.0)
		Malignant neoplasms of digestive organs	287 (9.2)
		Other malignant neoplasms	301 (9.7)
	Diseases of the nervous system		1162 (37.4)
		Amyotrophic lateral sclerosis	88 (2.8)
		Parkinson’s disease	646 (20.8)
		Epilepsy	510 (16.4)
		Multiple system atrophy	66 (2.1)
		Other diseases of the nervous system	26 (0.8)
	Pneumonia		878 (28.3)
		Bacterial pneumonia	41 (1.3)
		Pneumonia, organism unspecified	284 (9.1)
		Pneumonitis due to solids and liquids	615 (19.8)
		Other pneumonia	8 (0.3)
	Dementia		1149 (37.0)
	Cerebrovascular disease		1249 (40.2)
	Respiratory failure		302 (9.7)
	Severe motor and intellectual disabilities		1 (0.0)
	Cerebral palsy		18 (0.6)
Institution where gastrostomy was performed	Hospital		3078 (99.1)
	Clinic		18 (0.6)
Evaluation of swallowing function additionally calculated at the time gastrostomy was performed	Yes		704 (22.7)
	No		2401 (77.3)

Note: Some patients had more than one disease.

**Table 2:** Information on medical institutions that provided home semi-solid enteral nutrition guidance and management (N = 1405).

First Medical Institution That Provided HSEN	Second Medical Institution That Provided HSEN	n (%)
Hospital	Hospital	257 (18.3)
Hospital	Clinic	130 (9.3)
Clinic	Hospital	3 (0.2)
Clinic	Clinic	719 (51.2)
Hospital	No calculation	237 (16.9)
Clinic	No calculation	45 (3.2)
Unknown		14 (1.0)

HSEN, Home Semi-solid Enteral Nutrition Guidance and Management.

**Table 3:** Medical care and other patient characteristics during the period in which home semi-solid enteral nutrition guidance and management was being provided (N = 1405).

		n (%)
Prescription of RACOL-NF Semi-Solid for Enteral Use	Yes	1202 (85.6)
	No	203 (14.4)
Measurement of serum selenium levels	Yes	3 (0.2)
	No	1402 (99.8)
	No. of times performed (n = 3), median (min–max)	1 (1–1)
Home-visit rehabilitation guidance and management received by home-care patients	Yes	3 (0.2)
	No	1402 (99.8)
	No. of sessions (n = 3), median (min–max)	1 (1–12)
Home-visit dental care received	Yes	520 (37.0)
	No	885 (63.0)
	No. of sessions (n = 520), mean (SD)	6.8 (3.8)
Home-visit dental hygiene guidance received	Yes	24 (1.7)
	No	1381 (98.3)
	No. of sessions (n = 24), mean (SD)	3.1 (3.3)
Home-care patient home-visit oral rehabilitation guidance and management received	Yes	15 (1.1)
	No	1390 (98.9)
	No. of sessions (n = 15), mean (SD)	3.5 (2.9)
Death	Yes	5 (0.4)
	No	1400 (99.6)

Note: Data are presented as mean (SD), except for variables with small numbers of observations, which are presented as median (min–max). SD, standard deviation; min, minimum; max, maximum.

**Table 4:** Medical care and other patient characteristics after the end of home semi-solid enteral nutrition guidance and management (N = 707).

		n (%)
Prescription of RACOL-NF Semi-Solid for Enteral Use	Yes	581 (82.2)
	No	126 (17.8)
Measurement of serum selenium levels	Yes	7 (1.0)
	No	700 (99.0)
	No. of times performed (n = 7), median (min–max)	1 (1–2)
Home-visit rehabilitation guidance and management received by home-care patients	Yes	1 (0.1)
	No	706 (99.9)
	No. of sessions (n = 1), median (min–max)	2 (2–2)
Home-visit dental care received	Yes	323 (45.7)
	No	384 (54.3)
	No. of sessions (n = 323), mean (SD)	8.3 (3.9)
Home-visit dental hygiene guidance received	Yes	40 (5.7)
	No	667 (94.3)
	No. of sessions (n = 40), mean (SD)	6.1 (3.8)
Home-care patient home-visit oral rehabilitation guidance and management received	Yes	15 (2.1)
	No	692 (97.9)
	No. of sessions (n = 15), mean (SD)	7.5 (4.6)
Death	Yes	12 (1.7)
	No	695 (98.3)

Note: Data are presented as mean (SD), except for variables with small numbers of observations, which are presented as median (min–max). SD, standard deviation; min, minimum; max, maximum.

**Table 5:** Changes in the length of hospital stay and length of hospital stay for pneumonia (N = 707).

	During the HSEN Provision Period, Mean (SD), Median (Q1–Q3)	After the End of HSEN Provision, Mean (SD), Median (Q1–Q3)	Change, Mean (SD), Median (Q1–Q3)	*p* Value (Shapiro-Wilk Test)	*p* Value (Wilcoxon Signed-Rank Test)
Length of hospital stay (days)	8.2 (21.3) 0 (0–3)	29.1 (64.0) 0 (0–29)	20.9 (65.2) 0 (0–20)	<0.001	<0.001
Length of hospital stay (days/month)	1.74 (5.25) 0.00 (0.00–0.61)	2.39 (5.26) 0.00 (0.00–2.38)	0.66 (7.23) 0.00 (0.00–1.32)	<0.001	<0.001
Length of hospital stay for pneumonia (days)	3.1 (10.8) 0 (0–0)	10.2 (25.4) 0 (0–0)	7.1 (26.6) 0 (0–0)	<0.001	<0.001
Length of hospital stay for pneumonia (days/months)	0.58 (2.74) 0 (0–0)	0.84 (2.09) 0 (0–0)	0.26 (3.36) 0 (0–0)	<0.001	<0.001

HSEN, Home Semi-solid Enteral Nutrition Guidance and Management. SD, standard deviation. Q1–Q3, first to third quartiles.

## Data Availability

The datasets used in this study were obtained from DeSC Healthcare, Inc. Owing to legal, ethical, and contractual restrictions related to the medical information contained in the datasets, the data are not publicly available. Access to these data may be granted by DeSC Healthcare, Inc. upon reasonable request and subject to their approval.

## Supplementary Materials

The following supporting information can be downloaded at: https://www.mdpi.com/article/10.3390/nu18152442/s1, Figure S1: Number of home semi-solid enteral nutrition guidance and management sessions by disease; Table S1: Patient characteristics by disease; Table S2: Reasons for hospitalization.

## Abbreviations

The following abbreviations are used in this manuscript:

SEN	semi-solid enteral nutrition
HSEN	home semi-solid enteral nutrition guidance and management
FY	fiscal year
