# Supplementary material for: Clinical Reality and Outcomes of Home Semi-Solid Enteral Nutrition Guidance and Management Using a Medical Claims Database in Japan: A Retrospective Cohort Study
Source: Nutrients. 2026 Jul 27;18(15):2442. doi: 10.3390/nu18152442 (PMC13467615; doi:10.3390/nu18152442)
Supplement: Supplementary file 1 [file nutrients-18-02442-s001.zip › nutrients-4354589-supplementary.pdf]

Supplementary Table S1. Patient characteristics by disease (N = 1405).

		Malignant neoplasm n (%)	Nervous system diseases n (%)	Pneumonia n (%)	Dementia n (%)	Cerebrovascular disease n (%)	Respiratory failure n (%)	Severe motor and intellectual disabilities n (%)	Cerebral palsy n (%)
Sex	Male (n = 658)	106 (65.4)	277 (49.6)	214 (56.9)	208 (36.9)	304 (48.5)	56 (52.8)	1 (100.0)	7 (58.3)
	Female (n = 747)	56 (34.6)	281 (50.4)	162 (43.1)	355 (63.1)	323 (51.5)	50 (47.2)	0	5 (41.7)
Age (years)	Mean (SD)	77.9 (10.9)	76.1 (12.3)	79.4 (11.5)	83.1 (7.1)	80.7 (9.3)	79.6 (12.5)	20.0 (N/A)	36.3 (22.1)
Number of sessions	Mean (SD)	6.9 (4.4)	7.9 (4.4)	6.8 (4.7)	8.1 (4.4)	7.8 (4.5)	7.8 (4.3)	10.0 (N/A)	6.8 (4.4)

Note: Some patients had more than one disease. SD, standard deviation; N/A, not applicable.

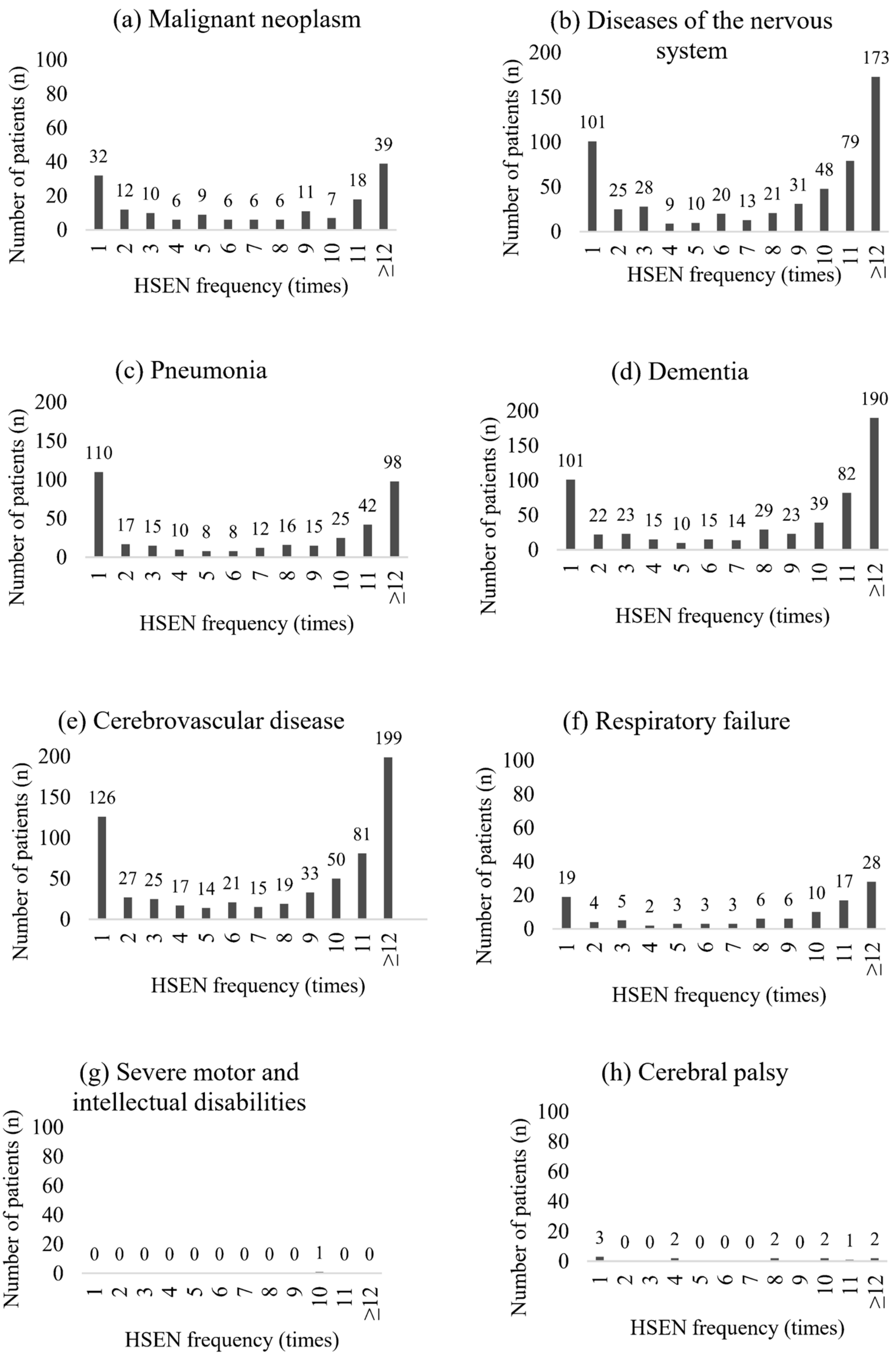

Supplementary Figure S1. Number of home semi-solid enteral nutrition guidance and management sessions by disease. Graph shows the number of patients according to the number of home semi-solid enteral nutrition guidance and management sessions received, by disease. HSEN, Home Semi-solid Enteral Nutrition Guidance and Management.

Supplementary Table S2. Reasons for hospitalization (N = 707).

Reason for hospitalization during the HSEN provision period	Hospitalized N = 224 n (%)	Reason for hospitalization after the last HSEN session	Hospitalized, N = 340 n (%)
Nervous system disease	91 (40.6)	Nervous system disease	143 (42.1)
Pneumonia	96 (42.9)	Pneumonia	194 (57.1)
Dementia	71 (31.7)	Dementia	128 (37.6)
Cerebrovascular disease	82 (36.6)	Cerebrovascular disease	141 (41.5)
Respiratory failure	36 (5.1)	Respiratory failure	81 (11.5)

HSEN, Home Semi-solid Enteral Nutrition Guidance and Management.
Note: Some patients had more than one disease.
